# Books Review

**Published:** 1869-11

**Authors:** 


### Books Review.

*The Mechanism of Dislocation and of Fracture of the Hip. With the Reduction of the Dislocations by the Flexion Method.* By HENRY J. BIGELOW, M. D. Philadelphia: HENRY C. LEA, 1869.

This is the most valuable contribution, to our knowledge, of the injuries in question. The whole subject is presented and illustrated in unsurpassed manner; and the views of the author, as presented and explained, will carry conviction wherever attentively considered. This work is invaluable; and every surgeon who has had any experience in the care of these injuries will peruse it, again and again, with the greatest satisfaction. The illustrations of the manner in which reduction of dislocation of the hip can be made by manipulations are exceedingly beautiful and instructive, and the general style of the publication of the work is unusually attractive. It is a subject of so great importance that we quote the author's

ABSTRACT :

1st. The anterior part of the capsule of the hip joint is a triangular ligament of great strength, which, when well developed, exhibits an internal and external fasciculus, diverging like the branches of the inverted letter Y. It rises from the anterior inferior spinous process of the ilium, and is inserted into nearly the entire length of the anterior intertrochanteric line.

2d. The Y ligament, the internal obturator muscle, and the capsule subjacent to it, are alone required to explain the usual phenomena of the regular luxations.

3d. The regular dislocations are those in which one or both branches of the Y ligament are unbroken; and their signs are constant.

4th. The irregular dislocations are those in which the Y ligament is wholly ruptured; and they offer no constant signs.

5th. In the regular dislocations of the hip, the muscles are not essential to give position to the limb, nor desirable as aids in its reduction.

6th. The Y ligament will alone effect reduction and explain its phenomena, a part of those connected with the dorsal dislocations excepted.

7th. During the process of reduction, this ligament should be kept constantly in mind.

8th. The rest of the capsule, except perhaps that portion beneath the internal obdurator tendon, need not be considered in reduction, if the capsular orifice is large enough to admit the head of the femur easily.

9th. If the capsular orifice is too small to allow easy reduction, it should be enlarged.

10th. The capsular orifice may be enlarged at will, and with impunity, by circumduction of the flexed thigh.

11th. Recent dislocations can be best reduced by manipulations.

12th. The basis of this manipulation is flexion of the thigh.

13th. This manipulation is efficient, because it relaxes the Y ligament, or because that ligament, when it remains tense, is a fixed point, around which the head of the femur revolves near the socket.

14th. The further manipulation of the flexed thigh may be either by traction or rotation.

15th. The dorsal dislocation owes its inversion to the external branch of the Y ligament.

16th. The so-called ischiatic dislocation owes nothing whatever of its character, or its difficulty of reduction by horizontal extension, to the ischiatic notch.

17th. "The ischiatic dislocation" is better named *"dorsal below the tendon,"* and is easily reduced by manipulation.

18th. The flexion of the thyroid and downward dislocations is due to the Y ligament, which, in the first, also everts the limb, until the trochanter rests upon the pelvis.

19th In the pubic dislocation, the range of the bone upon the pubes is limited by this ligament, which, in the sub-spinous dislocation also, binds the neck of the femur to the pelvis.

20th. In the dorsal dislocation with eversion, the outer branch of the Y ligament is ruptured

21st. In the anterior oblique luxations, the head of the bone is hooked over the entire Y ligament, the limb being then necessarily oblique, everted and a little flexed.

22d. In the supra-spinous luxation, the head of the femur is equally hooked over the Y ligament, the external branch of which is broken. The limb may then remain extended.

23d. In old luxations, the period during which reduction is possible is determined by the extent of the obliteration of the socket, the strength of the neck of the femur, add the absence of osseous excrescence.

24th. Old luxations may possibly require the use of pulleys, in order by traction to avoid any danger which might result to the atrophied or degenerated neck of the bone from rotation.

25th. Right-angled extension, the femur being flexed at a right angle with the pelvis, is more advantageous than that which has usually been employed.

26th. To make such extension most effective, a special apparatus is required.

### FRACTURES OF THE NECK OF THE THIGH-BONE.

1st. The terms intra- and extra-capsular, applied to these fractures, have little practical significance.

2d. When a fracture near the head of the femur shows bony union, it is often impossible to say whether such a fracture was originally inside or outside of the capsular ligament.

3d. These fractures are therefore better divided, for practical purposes, into: 1st, the impacted fracture of the neck into the trochanter; 2d, other fractures of the neck.

4th. In this impacted fracture, the limb is everted, because the posterior cervical wall is almost always impacted, the anterior very rarely, and in a less degree.

5th. These conditions mainly result from the relative thickness of the two walls.

6th. While eversion is due to the rotation of the fractured bone on a hinge formed in the anterior cervical wall, shortening is generally due to the obliquity of this hinge.

7th. In a well-formed bone, the posterior and thin surface of the neck of the femur is prolonged into the cancellous structure beneath the intertrochanteric ridge and is the true neck

8th. The posterior intertrochanteric ridge is a buttress built upon the true neck, by which, when impacted, this ridge is sometimes split off.

---

*A Handy-Book of Ophthalmic Surgery for the use of Practitioners.* By John Z. Laurence. Philadelphia: Henry C. Lea, 1869.

The Handy-Book of Ophthalmic Surgery is well described by its name, for the effort to bring the principles and practice of modern Ophthalmic Surgery within a small compass has been most signally successful. The very busy practitioner, with neither time or opportunity to read the larger and more complete works upon Ophthalmology can, in this, find a condensed and satisfactory description of the present science and practice of Ophthalmic Surgery. We have complete chapters upon the following subjects, which really include the whole field of practice in this department: "Methods of examining the eye; general remarks upon Ophthalmic operations; diseases of the orbits, of the eye-lids and eye-lashes, of the lachrymal apparatus, of the muscles of the eye; injuries of the eye and orbit; diseases of the conjunctiva, of the sclerotic, of the cornea, of the iris and ciliary body, of the crystalline lens; amaurosis and amblyopia; glaucoma; diseases affecting the whole eye-ball; on vision; optical defects of vision." To which is added a complete index.

In looking over the whole work, we see that it is most admirably adapted to practical men, and to the wants of medical students who desire to follow lecturers by reading some standard and reliable author. It is fully up with the present views in Ophthalmology, and supplies, in a most desirable form, all that can be desired by the general practitioner, and by medical students.

---

*Physical Culture in Amherst College.* By Nathan Allen, M. D., Lowell, Mass.

At the request of the board of trustees of the above named institution, the author, one of their number, presented, at their last annual meeting, this report of the introduction, plan, history, results, advantages and importance of the system of physical culture which they have in charge. As being a thorough resume of a system of gymnastic training which is thoroughly successful in its operation, this report is of wider interest than its title would indicate. The propriety and necessity of the physical and hygienic education of students in educational schools is well recognized, while efforts in this direction have been, to a great extent, failures. The success of the system which is described, we attribute to the relative rank of the professorship and true importance of the department being recognized both officially and practically; and again, also, to the whole-souled, energetic character of him who is placed in immediate charge of the department. Says the author:—"In an institution where a large body of students require daily exercise, with as little exposure and loss of time as possible, the lighter gymnastics, as here practiced, are undoubtedly best adapted to effect the object designed, and accordingly the light gymnastics are conducted after the manner of military drill, for which all are required to appear, while a portion of the time is also spent in voluntary exercise on the heavy apparatus." The design is, that all the muscles of the body should be exercised in a manner to equalize best the circulation of the blood—to expand the lungs—to aid the stomach in the digestion of food—to strengthen the joints—develope all parts of the body in harmony with the most efficient action of the brain. We should like to have seen stated an opinion as to the compatibility of hard mental labor, and the same degree of physical exertion, as derived from the author's experimental observation. Prof. Hitchcock's carefully recorded statistics in the appendix of the report sustain the general observations respecting the salutary effects of the system, to the inspection of which we invite any who are concerned in a matter of present and future importance.

---

*Carbolic Acid. Its action and uses.* By CHAS. F. J. LEHLBACH, M. D., Newark, N. J.

In this report, which is reprinted from the Transactions of the Medical Society of New Jersey, are presented the results of the author's own experience in the use of this agent, the cases of which are thus enumerated: "As a dressing to wounds; in carbuncles; in conjunctivits; burns and scalds; in dissecting wounds and pustules; in various species of impetigo, mentagra, scabies, gonorrhoea, chancre, leucorrhoea, herpes, diphtheria, diarrhoea in children and the sickness of pregnancy." Carbolic acid, notwithstanding the prejudice which it had to encounter as an innovator, has established itself as of real value to the profession. In surgical dressings it acts as a deodorizer, stimulant and anti-zymotic; and its use has been followed by marked results, but its value, when used internally, is not so well demonstrated. It is quite probable that creosote would have accomplished the same results as this agent now does, had not its odor limited its use.

---

*Hygiene in its relations to Therapeusis.* By ALFRED L. CARROLL, M. D.

In this work the author directs attention to the possible application of hygienic agents to special pathological indications. "Many maladies formerly supposed to demand the most energetic interference of remedial art, are now known to be self-limiting, with a natural tendency to termination in health, and not capable of being shortened or materially modified by the administration of drugs; and it seems probable that all so called acute diseases may be classed in this category. In these, and in the treatment of diseases generally, many, perhaps most enlightened physicians of the present day, recognize the subordination of pharmaceutic agents to hygienic influences." The author, as one of the editors of the *New York Medical Gazette*, has had ample opportunities of observation respecting the true progress of medical science.

---

*A Course of Practical Chemistry arranged for the use of Medical Students.* By WM. ODLING, M. D., F. R. S. *From the Fourth revised London edition.*

This work is a valuable manual of qualitative analysis for the use of students in laboratory and office practice. The author is a chemist of eminence, who is at present preparing a large and valuable work on chemistry, which is being published in London. The first chapter of this book gives an introductory description of chemical reactions and manipulations; the second chapter presents the regular method of examination for, and individual detection of, bases and their groups, and of the acids; chapter third embraces toxicological examination; the last chapter is on animal chemistry, of which the part on urinary examination is espeeially simple, systematic, and determinative.

---

*Proceedings of the Texas State Medical Society.*

The convention for organizing the Texas State Medical Society was held in the city of Houston, June 15th; and, on the two succeeding days, the Society adopted a constitution, by-laws, and the code of ethics of the American Medical Association. These are published in full in the report of the proceedings, which have been extensively distributed throughout the State.

---

*Annual Address delivered by* W. O. BALDWIN, M. D., *before the American Medical Association.*

The introductory part of the address is quite excellent in tone, and is well calculated to establish good-will among the members of the profession. It is very proper, also, that the attention of the Association should have been thus directed towards, and the efforts of its members enlisted in, the attainment of a higher standard of education for the medical profession of our country. It is to be hoped that every such effort will be crowned with new success.

---

*Fœticide or Criminal Abortion.* By HUGH L. HODGE, M. D.

A lecture introductory to the course in Obstetrics and Diseases of Women, relating to the criminality of Foeticide is here presented by the author, in a convenient form for general circulation.

*Oregon Medical and Surgical Reporter.* Edited by E. R. Fiske, A. M., M. D., Salem, Oregon.

We are happy to welcome this new contribution to current medical literature and to our exchange list. The editor is the "Professor of Theory and Practice" in Willamette University, the Faculty of which institution act as his assistant editors' while a number of the prominent professional men of the State have accepted positions as collaborators. The spirit of the editorial address is good, and supported by such a professional corps, the journal cannot fail to be worthy of support.

---

*The Chicago Medical Journal* has been entrusted for publication to Messrs. Keen & Cooke, Nos. 113 and 115 State Street, Chicago, who have brought out the journal in new type and dress, in which it presents a very becoming appearance. The editors, Drs. J. A. Allen and Walter Hay, relieved of a complication of duties, will now devote themselves to editorial matters exclusively.

